# Is It About Speech or About Prediction? Testing Between Two Accounts of the Rhythm–Reading Link

**DOI:** 10.3390/brainsci15060642

**Published:** 2025-06-14

**Authors:** Susana Silva, Ana Rita Batista, Nathércia Lima Torres, José Sousa, Aikaterini Liapi, Styliani Bairami, Vasiliki Folia

**Affiliations:** 1 Psychology Department, Faculty of Psychology and Educational Sciences, University of Porto, 4200-135 Porto, Portugal; 2 Laboratory of Neuropsychology and Behavioral Neuroscience, School of Psychology, Aristotle University of Thessaloniki, University Campus, 54124 Thessaloniki, Greece

**Keywords:** Temporal Sampling Framework, reading, rhythmic skills, stress-timed rhythm, cross-linguistic comparison

## Abstract

Background/Objectives: The mechanisms underlying the positive association between reading and rhythmic skills remain unclear. Our goal was to systematically test between two major explanations: the Temporal Sampling Framework (TSF), which highlights the relation between rhythm and speech encoding, and a competing explanation based on rhythm’s role in enhancing prediction within visual and auditory sequences. Methods: We compared beat versus duration perception for their associations with encoding and sequence learning (prediction-related) tasks, using both visual and auditory sequences. We also compared these associations for Portuguese vs. Greek participants, since Portuguese stress-timed rhythm is more compatible with music-like beats lasting around 500 ms, in contrast to the syllable-timed rhythm of Greek. If rhythm acts via speech encoding, its effects should be more salient in Portuguese. Results: Consistent with the TSF’s predictions, we found a significant association between beat perception and auditory encoding in Portuguese but not in Greek participants. Correlations between time perception and sequence learning in both modalities were either null or insufficiently supported in both groups. Conclusions: Altogether, the evidence supported the TSF-related predictions in detriment of the Rhythm-as-Predictor (RaP) hypothesis.

## 1. Introduction

Time perception may be based on either beat or duration [1,2]. In beat-based time perception, time-related information is extracted with reference to a regular time unit (the beat). In duration perception, the absolute duration of time intervals is the target [3]. In the last few years, associations between reading performance and time perception have been widely reported. Some studies refer to duration perception [4,5,6,7], but most highlight beat-based time (rhythm) perception [8,9]. The idea of a rhythm–reading link is supported by findings of poor rhythm perception in dyslexics compared to typical readers [10,11,12], independent of general cognitive abilities [13]. The benefits of rhythm-based intervention for children with reading difficulties strengthen the rhythm–reading link further [14].

### 1.1. Encoding and Sequence Learning as Reading Components

The mechanisms underpinning the rhythm–reading link are yet to be determined. Most attempts to explain why beat-based time perception relates to reading suggest that rhythmic skills enhance reading-related abilities [10], such as phonological awareness [15,16,17] and rapid naming [18]. However, these abilities are actually tasks that engage multiple cognitive skills. For instance, Rapid Automatized Naming (RAN) is a highly complex, multidimensional task that engages visual, phonological, and articulatory skills [19]. Phonological awareness requires both phonological encoding and phonological working memory [20,21]. Therefore, instead of investigating associations between reading and any of these complex tasks, a better option could be to adopt a working model of elementary reading components, which is closer to the idea of cognitive processes.

One possibility is to consider two basic sequencing processes—(1) encoding of basic units and (2) sequence learning—for the two modalities involved in reading: audition and vision. Reading (print-like, visual, static sequences) must be preceded by the acquisition of speech-like (auditory, temporal sequences) processing skills. Both print and speech processing demand the ability to create internal representations of basic units, such as phonemes and graphemes (encoding), as well as the ability to extract sequential regularities, such as frequent phoneme or grapheme combinations, in a given language (sequence learning). The literature shows that these components are affected in dyslexia beyond the linguistic domain [22], namely in statistical learning for auditory sequences [23], visual encoding [24], and visual artificial grammar learning [25]. Therefore, going beyond the conceptual level (see below for the integration in current frameworks), the relation of reading skills with encoding and sequence learning is backed by empirical evidence. This allows us to look at these reading components as proxies of reading skills.

### 1.2. Hypothesis-Dependent Associations of Rhythm Skills with Reading Components

The association of beat processing with encoding and sequence learning is supported by evidence. It is known that the temporal organization of units—auditory units or visual units presented one at a time according to a regular beat—facilitates encoding [26,27], as well as the extraction of sequential regularities [28]. Therefore, it is reasonable to expect that beat-related skills enhance these two sequencing processes in the auditory domain for beat-like inputs such as speech. Regarding visual, non-temporally organized materials (equivalent to print), research has not, to our knowledge, examined the potential association of beat skills with encoding or sequence learning.

Criticality, different associations between beat perception and sequencing processes are predicted by the two currently available and competing hypotheses for the rhythm–reading link. The first one (Hypothesis 1) is part of the Temporal Sampling Framework (TSF [22]). According to TSF, the brain oscillatory correlates of beat perception would be responsible for the effective encoding of speech at multiple levels (phonemes, syllables, and stress accents), and this might improve sequence learning for speech. As oscillatory activity in the brain synchronizes with (entrains to) incoming speech units at the relevant frequency bands (e.g., delta oscillations below 2 Hz, synchronizing with incoming sequences of stress accents), the brain “knows when” each new unit will arrive. Attention to speech units increases, encoding is enhanced, and this may also improve the extraction of sequential regularities. In sum, efficient entrainment to beat-based auditory input (musical rhythm) would correlate with efficient speech processing—the basis of reading skills. Consistent with this, dyslexics have shown atypical entrainment to low frequencies in the auditory domain [22].

TSF is the underlying framework for most studies that suggest a mediating role of phonological awareness in the rhythm–reading link [10,16]. Although significant results for a mediation of this sort can be found [17], null results have also been reported [15], casting doubt on the rhythm–auditory encoding–reading chain proposed by TSF. An alternative path linking beat perception with reading components has, thus, been proposed (Hypothesis 2). According to [5], the path would be that individuals who are skilled in temporal prediction (beat perception, “knowing when”) are expert general predictors that are also experts in ordinal prediction (“knowing what” comes next—which letter, which sound). Sequence learning is necessary for efficient ordinal prediction, as the latter is fed by knowledge of frequent sequential patterns. From this viewpoint, rhythm skills would foster reading, not via encoding but instead by enhancing domain general (auditory or visual) sequence learning abilities.

In sum, according to Hypothesis 1 (TSF), rhythm perception should relate to auditory encoding and possibly auditory sequence learning, while, under Hypothesis 2 (Rhythm-as-Predictor hypothesis (RaP)), it should relate to both auditory and visual sequence learning. In other words, associations of rhythm skills with auditory encoding but not visual sequence learning would contribute to rule out Hypothesis 2, with the reverse applying to Hypothesis 1 (rhythm skills associated with visual sequence learning but not auditory encoding). Though evidence in favor of Hypothesis 1 is available, little or nothing is known about the competing RaP hypothesis, and much less about the outcomes of testing one against the other.

### 1.3. The Complementary Role of Speech Rhythm and Rhythmic Regularity in Hypothesis Testing

A complementary way of testing TSF vs. the RaP hypothesis is to investigate the relationship between rhythm skills and auditory sequencing processes across languages with different patterns of speech rhythm. The rhythm of each language may be classified along a continuum, where syllable timing (similar time intervals across syllables) and stress timing (similar time intervals across stress accents, with significant variations in syllable length) are at the poles. English has been predominantly classified as a stress-timed language. In Greek, it is more common to have stable syllable duration even for the unstressed [29]. More recent evidence [30,31,32] has strengthened this claim by showing that Greek readers process rhythmically stressed syllables similarly to unstressed ones and identify unstressed syllables better than English participants. As for (European) Portuguese, it is believed to have at least a noticeable marked distinct stress-timed component [33,34,35], namely high ∆C values (variability in duration of consonantal intervals) that characterize other stress-timed languages like English, Polish, or Dutch [33]. Therefore, stress seems to be more present in Portuguese than in Greek. 

How might these rhythm-related cross-language differences modulate the relationship between rhythm and reading mechanisms? The answer relates to the fact that the “average” beat in music (500 ms, 120 bpm) is within the delta range (1–4 Hz, beat lengths between 1000 and 250 ms), which characterizes stress rhythm. In contrast, the syllable rate is around 6 Hz, 167 ms per syllable, which is too fast for a musical beat (360 bpm) and thus unrelated to musical rhythm. According to the author of the TSF, stress rhythm is the most important target of entrainment in any language, as it would be the structure within which faster, syllable-, and phoneme-related frequencies are nested [36]. However, it is reasonable to claim that speakers of syllable-timed languages like Greek should not benefit from music-like, delta-related beat skills to enhance their entrainment to speech [37] as much as speakers of stress-timed languages. Supporting this claim, cross-language research has shown that stress-timed languages elicit stronger entrainment between 1 and 4 Hz—delta band, the frequency of stress accents [33,38]—than syllable-timed languages. Critically, it showed that Greek participants’ association between beat perception and phonological processing (expected to improve with delta band entrainment) is nonexistent [39].

We also manipulated the regularity of time intervals in auditory sequences. We did it because, whatever the language, speech is never strictly regular or periodic (i.e., isochronous), and the two hypotheses under testing make different predictions based on this. The TSF is not explicit about differences in oscillatory entrainment to strictly periodical vs. aperiodic (speech-like) information, but it assumes that speech may “work like music” and be a target of entrainment despite its irregularity [7,40,41,42]. In contrast, the competing RaP hypothesis [5] proposes that beat perception is not akin to the aperiodic structure of speech, and thus, duration perception would be a more plausible enhancer of speech encoding. To capitalize on these differences and expand our testing tools, we tested the associations of both beat and duration perception with two versions of auditory encoding and sequence learning: periodic vs. aperiodic.

In summary, in the present paper, we examined the associations of rhythm perception skills with the four reading components we considered: auditory (temporal) encoding, visual (static) encoding, and auditory and visual sequence learning in native Portuguese vs. Greek speakers. Our goal was to perform a systematic test between TSF and its competitor (rhythm as a prediction enhancer) as explanatory mechanisms of the rhythm–reading link. Significant links between rhythm perception and auditory encoding, plus an increased importance of beat for auditory encoding in Portuguese compared to Greek speakers, would favor the TSF. Significant links between rhythm perception and all types of sequence learning, as well as null evidence of cross-language differences in the relation between rhythm and auditory encoding skills, would favor the second hypothesis.

Finally, if rhythm perception relates to auditory encoding, as predicted by the TSF, and the irregularity of speech is tolerable under the entrainment mechanism, we should see no differences between periodic and aperiodic encoding/sequence learning when it comes to their relationship with beat skills. In contrast, and according to the RaP approach, aperiodic encoding/sequence learning would be more closely related to duration than beat perception.

## 2. Materials and Methods

### 2.1. Participants

An a priori power analysis for zero-order correlations showed that we would need a sample of between 24 and 67 participants to capture a medium effect size with 80% power and an alpha error probability of 0.05. To capture a small correlation, we would need a sample size between 68 and 616. A total of 209 participants (148 Portuguese and 61 Greek native speakers) took part in the study, all recruited from university courses.

Portuguese (114 female, 25 male, 1 non-binary, and 8 participants with missing sociodemographic data) and Greek participants (55 female, 6 male) had similar mean ages (*p* = 0.84; Portuguese: M ± SD = 20.8 ± 5.10; Greek: M ± SD = 20.6 ± 1.79) and similar years of music education or music practice (*p* = 0.67; Portuguese: M ± SD = 2.40 ± 3.31; Greek: M ± SD = 2.18 ± 3.44). However, there were significant differences between the two groups of participants regarding years of schooling (*t*(199) = −6.36, *p* = < 0.001, d = 0.879): Portuguese participants had an average of 12.9 years of formal education (SD = 1.98), and Greek participants had 14.51 years (SD = 1.51). None of the participants reported a known history of neurodevelopmental or psychiatric disorders, nor any known history of auditory impairment. All participants gave their informed consent according to the Declaration of Helsinki. The project was approved by the Ethics Committee from the Faculty of Psychology and Educational Sciences at the University of Porto (Ref. 2023/03-13) and the Research Ethics Committee of the Faculty of Psychology at Aristotle University of Thessaloniki (Ref. 67β/23-01-2023).

### 2.2. Stimulus Materials

**Figure 1:**
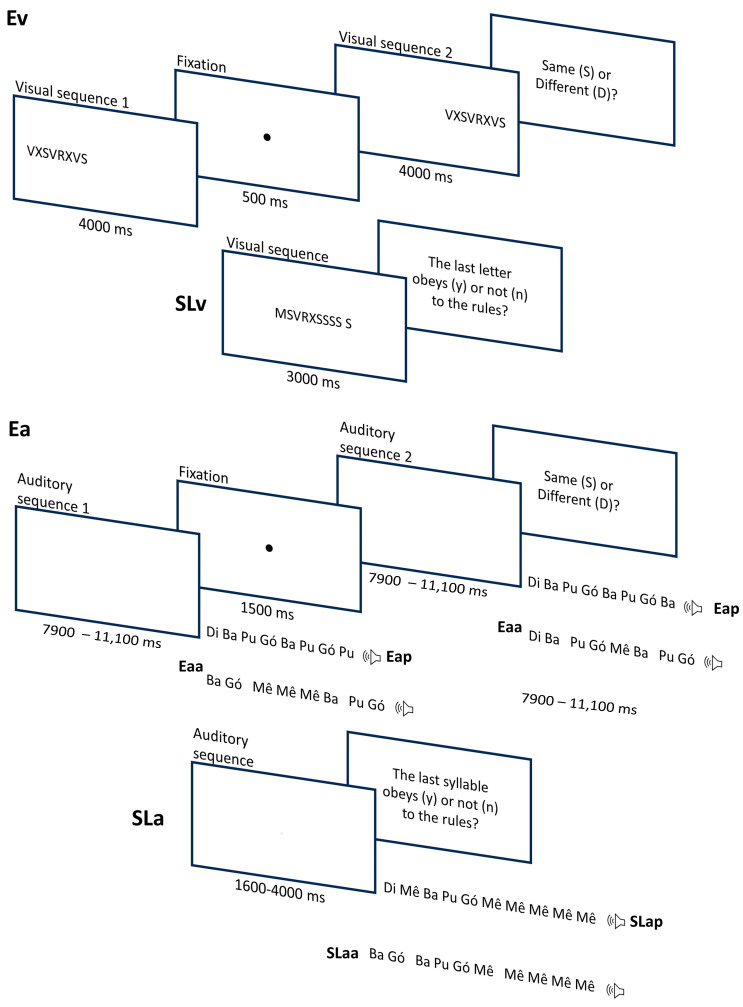
Stimuli and experimental paradigm for the sequencing tasks. Note: ms = milliseconds; Ev = Encoding visual stimuli; SLv = Sequence Learning with visual stimuli; Ea = Encoding of auditory stimuli; Eaa = Encoding of auditory aperiodic stimuli; Eap = Encoding of auditory periodic stimuli; Sla = Sequence Learning with auditory stimuli; SLaa = Sequence learning with auditory aperiodic stimuli; SLap = Sequence Learning with auditory periodic stimuli.

To assess participants’ ability to encode visual stimuli (Ev; see Figure 1), we used 24 pairs of letter sequences (five different letters: V, M, X, S, and R; sequence lengths ranging from 8 to 12 letters; see the Supplementary Materials) retrieved from [43]. Half of these pairs were made up of the same sequence, and the other half consisted of different sequences. All sequences were formed according to a common set of rules, or grammar. In the Sequence Learning task with visual stimuli (SLv), we used 24 sequences with the same five letters (V, M, X, S, and R). Each sequence ranged between 4 and 10 letters (see the Supplementary Materials). In half of these sequences, the last letter obeyed the same rules that were used to generate encoding sequences (correct sequences), while, in the other half, the last letter violated those rules (incorrect sequences).

For the auditory tasks (Encoding of auditory periodic stimuli—Eap, Sequence Learning with auditory periodic stimuli—SLap, Encoding of auditory aperiodic stimuli—Eaa, and Sequence Learning with auditory aperiodic stimuli—SLaa), we used the same exact sequences (abstract combinations of units) but substituted the letters (V, M, X, S, and R) with CV syllables (ba, di, gó, mê, and pu). The five syllables were spoken by an adult Portuguese speaker (female) instructed to keep the pitch constant within and across vowels. The five syllables were recorded with a sampling rate of 44.1 kHz and later normalized for amplitude. We had two sets of sequences, depending on temporal structure: those with periodic syllable onsets (isochronous, constant inter-onset intervals across syllables) vs. those with aperiodic (non-isochronous) onsets. Participants completed the auditory encoding twice using these two sets (Eap for periodic and Eaa for aperiodic), and the same went for auditory sequence learning (SLap and SLaa). For the periodic versions, onsets occurred every 400 ms, and for the aperiodic ones, they varied between 350, 450, 550, and 600 ms (see the Supplementary Materials). The original auditory sequences that were used in previous studies had a periodic structure. Interval sequences for the aperiodic condition were, thus, generated during the preparation of the current study. In this process, the sequence of irregular intervals was controlled to match the duration of the corresponding periodic versions. Figure 1 illustrates the trial structures of Ev, SLv, Eap, SLap, Eaa, and SLaa.

**Figure 2:**
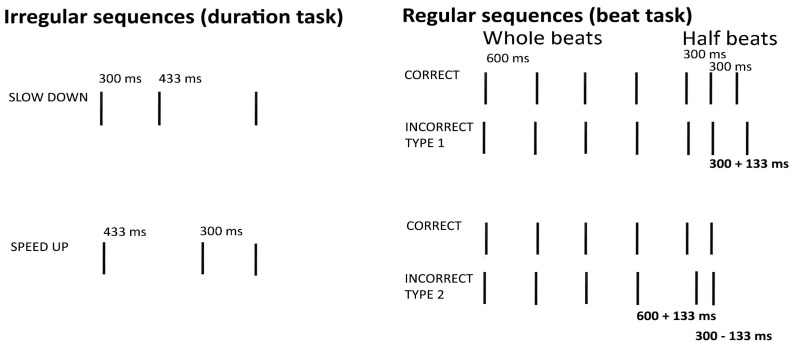
Stimulus structure for the duration (irregular) and beat perception (regular). Straight lines indicate beep onsets.

For the beat perception task, we used a set of eight sequences of beeps (always the same beep, 67 ms long, F0 = 450 Hz), created for a previous study of ours [44]. The sequences contained the equivalent to 7–10,600 ms beats, with lengths ranging between 4200 ms and 6000 ms. In the correct sequences, participants were presented with temporal sequences that began with whole beats (600 ms intervals) and ended with half beats (300 ms), maintaining an integer ratio of 1:2. The incorrect sequences included deviant intervals, which introduced non-integer ratios. This modification involved adding or subtracting 133 ms from the intervals. Specifically, in the first case, the last two onsets (stimuli 1–4) were altered, introducing a deviant 300 ms interval. In the second case, the two onsets before the final one (stimuli 5–8) were modified, introducing one deviant 600 ms interval and one deviant 300 ms interval (Figure 2).

For the duration perception task, we used 16 sequences of three beeps (same beep sound used in the beat perception task) separated by two intervals. These sequences were also designed for a previous study of our team [45]. Half of these sequences sped up (first interval longer than first), and the other half slowed down (second interval longer than first), with the two halves matched for the interval content (see the Supplementary Materials). A 200 ms silence preceded each sequence. The mono audio files had a bit depth of 16, 44.1 kHz sampling frequency, and lengths ranging between 467 and 933 ms (Figure 2).

### 2.3. Procedure

Data collection was carried out online through a link that provided access to the tasks and lasted around 1 h. The tasks were organized into five different blocks: (1) Encoding and Sequence learning with visual stimuli (Ev and SLv), (2) Encoding and Sequence Learning with auditory periodic stimuli (Eap and SLap), (3) Beat Perception Task, (4) Duration Perception Task, and (5) Encoding and Sequence Learning with auditory aperiodic stimuli (Eaa and SLaa). Counterbalancing was implemented to prevent critical learning, fatigue, or other confounding effects. In order to achieve a balance between control and a manageable number of experiment versions, we focused on critical contrasts such as that between periodic and aperiodic auditory tasks (2 and 5) and the one between beat and duration perception (blocks 3 and 4). Time perception tasks were always presented in the middle of the session to preserve them from strong order effects. As a result, half the participants went through the block sequence 1–2–[3–4]–5 (periodic first, beat first) and the other half through 1–5–[4–3]–2 (aperiodic first, duration first).

Participants received the instructions and completed one practice trial before the beginning of each task. Sequence presentation was randomized every time the experiment ran. In Encoding visual stimuli (Ev), participants saw a sequence of letters on the monitor (all letters presented at the same time for 4000 ms), followed by a 500 ms blank screen. Then, they saw a second sequence (also 4000 ms, with a total trial duration of 9500 ms) and were asked to judge if the two sequences were the same or different by pressing two different keys on the computer keyboard. After that, in the Sequence Learning with visual stimuli (SLv), participants saw a sequence on the screen for 3000 ms and had to judge if the last letter followed the pattern of the sequence or not by pressing two different keys.

The Encoding and Sequence Learning tasks with auditory periodic/aperiodic stimuli (Eap, SLap, Eaa, and SLaa) were similar to the Ev and SLv, differing only in that participant listened to syllable sequences (temporally organized) instead of seeing the full visual sequence on the computer screen. In the encoding task, the sequences in each pair were separated by 1500 ms of silence, and the trial had a total duration ranging between 7900 and 11,100 ms. The auditory sequence learning trials lasted between 1600 and 4000 ms.

In the beat perception task, participants were instructed to press one of two keys, depending on whether they judged the sequence was correct—i.e., followed a beat—or incorrect—i.e., did not follow a beat and sounded “like someone suddenly beginning to walk with a limp”. In the duration task, participants were asked to judge if sequences of three beeps were speeding up or slowing down by pressing two different keys. This task has been used in several studies from our team [45,46], with systematic evidence of above-chance performance without ceiling effects in neurotypical populations. The same applies to the beat perception task, which we used in various studies (e.g., [44,45]) with consistent results.

### 2.4. Statistical Analysis

We began the analysis by examining the relationship between experimental tasks (sequencing tasks—Ev, SLv, Eap, SLap, Eaa, and SLaa and time perception tasks—beat and duration perception) and sociodemographic variables (age, schooling, music education or music practice, and language). This was done to identify any variables that needed to be controlled for further analyses. To achieve this, correlational analyses and independent samples *t*-tests were conducted.

Next, we proceeded with the core analysis, investigating the relationship between encoding/learning and beat/duration perception for each language group separately. First, we compared the accuracy of the eight experimental tasks x language groups against chance to ensure the reliability of performance. Subsequently, we computed Pearson correlations of each sequencing task with beat vs. duration perception for each group. Additionally, Bayes Factors (BFs) with default priors were calculated for each correlation using JASP software (https://jasp-stats.org/; Version 0.16.0, accessed on 10 June 2025) to further investigate the strength of evidence in favor of the null vs. the alternative hypothesis. Following the heuristics provided by [47], BFs ranging between 1 and 3 were considered weak, 3 and 10 were moderate, 10 and 30 were strong, and values above 30 indicated very strong evidence in favor of the alternative hypothesis. Conversely, BFs below 1 indicated evidence in favor of the null hypothesis: BFs between 1 and 0.33 provided weak evidence, between 0.33 and 0.10 moderate, between 0.10 and 0.03 strong, and below 0.03 were considered very strong. Finally, we compared correlations for the two groups with Fisher Z tests. We validated differences between language groups when we had evidence of differences between correlations, and one was significant and supported by a BF (>1), while the other was not. When correlations did not differ, we assumed significant associations in both groups when present and no correlation if one group showed non-significant values. When the two correlations were non-significant, we did not run the test.

## 3. Results

### 3.1. Experimental Tasks and Sociodemographic Variables

As presented in Table 1, schooling and language were the only variables significantly related to the experimental tasks: schooling was weakly associated with Ev and Eaa (*r* < 0.30), and Greek showed significantly higher scores than Portuguese participants in all encoding and sequence learning tasks, except for SLv and SLaa. Neither beat nor duration perception showed associations with the sociodemographic variables.

**Figure 3:**
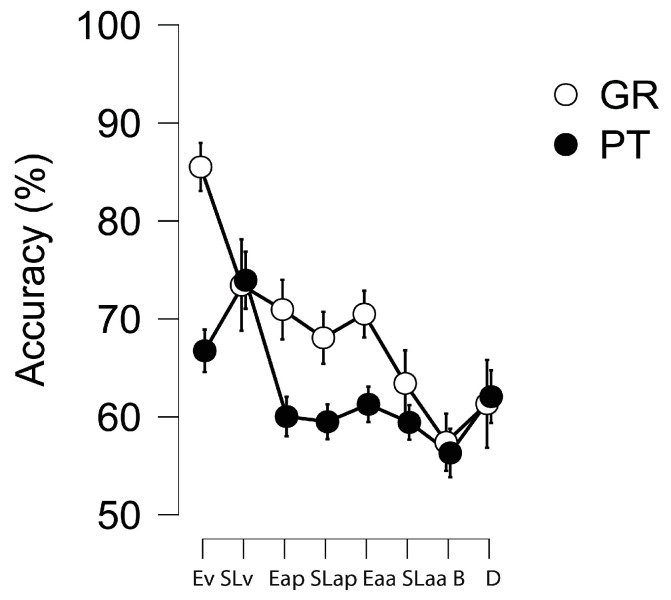
Portuguese (PT) vs. Greek (GR) participants’ performance in the experimental tasks. Note: Ev = Encoding visual stimuli; SLv = Sequence Learning with visual stimuli; Eap = Encoding of auditory periodic stimuli; SLap = Sequence Learning with auditory periodic stimuli; Eaa = Encoding of auditory aperiodic stimuli; SLaa = Sequence learning with auditory aperiodic stimuli; B = beat perception; D = duration perception.

One-sample *t*-tests showed above-chance performance levels (all *p* < 0.001) for all sequencing tasks in both groups (Figure 3).

Considering that Greek participants had higher levels of schooling (association between schooling and language; *t*(199) = −5.73, *p* = <0.001, *d* = 0.879; see Section 2.1), and that both schooling and language were related to Ev and Eaa, we conducted a mediation analysis to explore whether and to what extent the significant association between schooling and the two experimental tasks was mediated by language. The results on indirect effects revealed that language significantly mediates the relation between schooling and Ev; *B* = 1.70, *z* = 4.89, *p* < 0.001 (95% CI = 1.02–2.39), as well as that between schooling and Eaa; *B* = 0.76, *z* = 3.58, *p* < 0.001 (95% CI = 0.34–1.17). After removing the contribution of language, schooling had no effect on those variables (direct effects: *p* > 0.48 for Eva bd vs. *p* = 0.67 for Eaa), pointing to the presence of full mediation. Language was, thus, the only sociodemographic variable with an effect on sequencing tasks, eliminating the need to control for age, schooling, or music practice.

### 3.2. Correlations Between Sequencing and Time Perception Tasks per Language-Based Group

**Figure 4:**
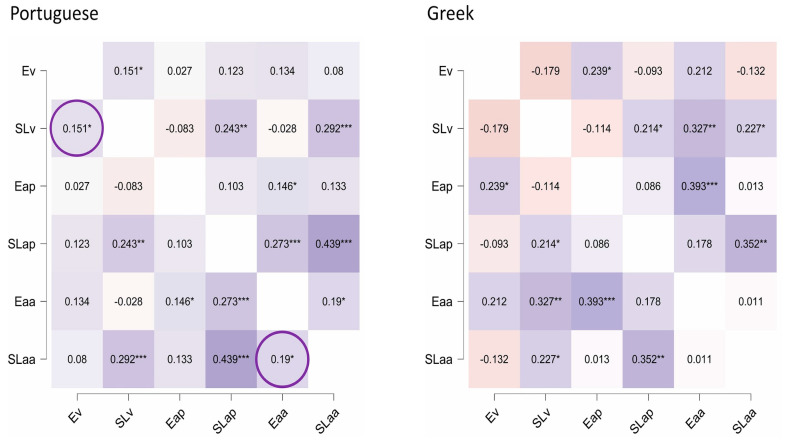
Correlations among the sequencing tasks. Note: Ev = Encoding visual stimuli; SLv = Sequence Learning with visual stimuli; Eap = Encoding of auditory periodic stimuli; SLap = Sequence Learning with auditory periodic stimuli; Eaa = Encoding of auditory aperiodic stimuli; SLaa = Sequence learning with auditory aperiodic stimuli. * *p* < 0.05, ** *p* < 0.001, and *** *p* < 0.0001.

The correlations between beat and duration perception were very weak and non-significant in both groups (Portuguese: *r* = −0.032, *p* = 0.648; Greek: *r* = 0.032, *p* = 0.403). Regarding correlations among sequencing tasks, these were more prominent among encoding tasks in the Portuguese group and among sequence learning tasks for the Greeks. Only Portuguese participants exhibited significant (though weak) correlations between encoding and sequence learning, specifically in the visual modality and aperiodic auditory tasks (Figure 4).

Regarding our main question, the correlations between beat perception and Eaa differed significantly across the language groups (Table 2). The Portuguese participants showed a significant moderate (positive) correlation with very strong Bayesian support, while the Greek participants displayed a non-significant value. Duration perception correlated positively with Eap in both groups, and the direct comparison showed non-significant differences. For Ev, SLap, and SLaa, we found weak correlations with beat in the Portuguese participants but direct comparisons (with no or weak Bayesian support) and non-significant values in the Greeks’ results. Following the principles we established, we did not endorse these correlations.

## 4. Discussion

Our goal was to perform a systematic test between the TSF and its competitor (Rhythm-as-Predicter enhancer) as explanatory mechanisms of the rhythm–reading link. Consistent with the TSF’s proposal, we found a significant association between beat perception and auditory encoding in Portuguese, but not Greek, participants. This pattern strengthens the TSF’s prediction that beat processing relates to auditory encoding. It is also consistent with the relevance of beat-like interval lengths (stress-timed, delta frequencies) in Portuguese but not Greek (which is syllable-timed). These cross-language differences were validated by a direct comparison between correlations. On the other hand, correlations of beat perception with visual sequence learning were null (moderate Bayesian evidence), and there was weak support for those established with auditory materials in the same task. Also, the potential role of duration perception in aperiodic auditory encoding (and, consequently, a correlation of it with duration) that could be expected based on the RaP hypothesis [5] was not supported (strong Bayesian support for the null hypothesis). These two findings speak against the role of ordinal prediction as a valid mechanism. Altogether, the evidence favored TSF-related predictions to the detriment of the Rhythm-as-Predicter enhancer hypothesis.

Despite the convergent evidence, we had a few unexpected results. One was the null association between beat skills and periodic encoding. According to the TSF’s predictions, beat skills should relate primarily with periodic (beat-like) sequences, but in order to favor speech encoding, they should also relate to aperiodic ones, showing some “tolerance” toward the irregularity of speech.

One way to make sense of this exclusion is to adopt another viewpoint and approach duration vs. beat as stimulus-driven, bottom-up vs. subject-driven, top-down processing, respectively [48]. Evidence shows that, typically, developing adults can efficiently detect regularities in irregular rhythmic patterns [49], indicating that finding the beat may be largely a top-down process. In the present study, it may have been the case that those with better beat skills (for 300–600 ms intervals) were able to enhance their performance on aperiodic (irregular, unpredictable) auditory encoding by imposing an internal beat as an organizer of the sequence events (400 ms inter-onset intervals). If we take into account that intervals between stressed words are not strictly regular in Portuguese, the possibility that beat skills help organize irregular (but not regular) sequences at these time scales (delta frequencies) would be consistent with our main hypothesis: Portuguese speakers, for whom (quasi-) periodicities in speech (stress accents) are closer to music-like beats, benefit from music-like beats (delta frequencies) as top-down organizers of incoming speech. In sum, the length of each of our “syllables” used in the auditory sequences may represent the length of the interval between stressed syllables in Portuguese due to the similarity in time scales (400 ms in syllables, 300–600 ms in beat). Since stress accents are not strictly periodic (though more than in Greek) and require top-down organization, beat skills (rather than duration skills) could be important for speech encoding in Portuguese. Simultaneously, as a process of stressing words in a regular rhythmic manner is incorporated in Portuguese (while, in Greek, each word is typically stressed via a specified lexical template), the proposed mechanism would be more critical (and more systematically employed) for successful encoding by Portuguese speakers.

Another unexpected link was that between duration and periodic auditory encoding. According to the TSF’s competitor hypothesis, an association with aperiodic encoding would make sense but not this one. How can we account for this result? One possibility is that duration skills were used to improve the identification of syllable onsets, which partly depend on phoneme-length perception. This interpretation is supported by several studies suggesting that sensitivity to duration and temporal structure supports auditory encoding strategies. These strategies are crucial for identifying syllable onsets and, consequently, for successful reading acquisition [7,40,41,42]. For example, Plourde et al. [7] found that children’s time-processing skills significantly predict their reading abilities, underscoring the importance of temporal perception in literacy development. Also, Andrade et al. [40] showed that children’s ability to process sequences in music—a skill that is related to duration sensitivity—predicts future reading proficiency. Similarly, Groth et al. and Steinbrink et al. [41,42] found that children with developmental dyslexia show deficits in processing temporal aspects, particularly vowel length. Since the syllable inter-onset interval was predictable here and, thus, not challenging, participants with better duration perception skills may have used their available capacity to improve syllable encoding via phonemic processing [50]. One way to address this limitation in future studies could be to use non-linguistic materials or materials which identity does not depend on length.

Another unexpected finding was the weak or absent relation between auditory encoding tasks and the respective sequencing tasks. Only aperiodic auditory encoding showed a weak link with auditory aperiodic sequencing, and this happened only in the Portuguese participants. Perhaps this encoding–prediction link is non-essential, and the time–reading link does not go much beyond efficient encoding.

One of the limitations of the current study is that, besides the experimental tasks, no other cognitive tests were administered, meaning that participants were not tested for reading or other cognitive skills. Therefore, the possibility that some of them may have undiagnosed reading- or cognitive-related difficulties leading to qualitative differences in functioning cannot be excluded, even though all of them were university students. Second, it is possible that memory skills played an important role in task performance and, thus, should be controlled for in further studies. Specifically, the fact that the auditory encoding tasks engaged skills other than encoding per se—namely, working memory—may have introduced confounds. Participants may have encoded accurately but forgotten the first sequence when they saw the second. The first limitation is easily addressed by evaluating participants’ cognitive skills before including them in the final sample, but the second would require other solutions. Concerning potential working memory-related confounds, one way of mitigating the problem would be replacing the same–different encoding task (based on two sequences) with a single-sequence task where, for instance, participants are asked to detect whether a particular subsequence is present or not. Another approach could be measuring participants’ working memory skills and controlling for these in the analysis.

Apart from our main goal, we found interesting evidence that may feed further studies or strengthen previous evidence. First, the Greek participants outperformed the Portuguese in almost all tasks. One hypothesis is that Greeks are less familiarized with Latin letters as conveyors of linguistic meaning and thus benefit from less interference (e.g., by forming acronyms as memory strategies). Second, the dissociation between beat and duration skills was quite clear, in line with some studies (e.g., [45]) but not necessarily a trivial finding.

Regarding suggestions for future studies on the rhythm–reading link, we would mention two. First, it is known that phonological awareness mediates the rhythm–reading relationship (also supported by the TSF [17]). Though phonological awareness is a complex task that engages several components, it includes auditory encoding; the manipulation of phonemes is not possible unless phonemic representations are available. In this sense, it could be important to add phonological awareness tests to our paradigm and correlate these with beat perception auditory encoding tasks. If it is true that our auditory encoding tasks represent phonological awareness, we should expect it to be associated with both auditory encoding (overlapping processes) and beat perception (both benefiting from it). This could provide an index of external validity to the present findings. Second, the TSF is based on the concept of entrainment, which is a neurophysiological phenomenon that must be captured with an EEG to be quantified. Here, we worked with behavioral manifestations of potentially different levels of entrainment, but a direct approach to brain oscillations while encoding visual and auditory materials is a challenge for future studies.

## 5. Conclusions

The current study investigated the association between reading and rhythmic skills and tested two major explanatory mechanisms: the TSF and the RaP hypothesis. We compared beat perception and duration perception associations with encoding and sequence learning tasks in both visual and auditory sequences between Portuguese and Greek participants. Our evidence favored the Temporal Sampling Framework as the more plausible mechanism for explaining the positive association between reading and rhythmic skills, particularly the connection between beat perception and auditory encoding in languages with a stress-timed rhythm like Portuguese.

## Figures and Tables

**Table 1:** Associations between accuracy in the experimental tasks and sociodemographic variables (*n* = 209).

	Age *r*	Schooling *r*	Music Education/Practice *r*	Language			
				*t*	*U*	*p*	Effect Size
Visual							
Ev	−0.054	0.175 *	0.032		8082.00	<0.001	0.622 ^a^
SLv	0.041	0.020	−0.104		4460.00	0.892	−0.079 ^a^
Auditory Periodic							
Eap	−0.082	0.074	0.035	5.663		<0.001	0.862 ^b^
SLap	−0.109	0.044	0.025		6302.50	<0.001	0.314 ^a^
Auditory Aperiodic							
Eaa	−0.019	0.153 *	−0.006		6566.00	<0.001	0.358 ^a^
SLaa	−0.045	−0.001	−0.085		5281.50	0.053	0.0134 ^a^
Beat	−0.050	0.053	0.056		4473.00	0.919	−0.007 ^a^
Duration	−0.079	−0.044	0.078		4341.50	0.665	−0.030 ^a^

Note: Ev = Encoding visual stimuli; SLv = Sequence Learning with visual stimuli; Eap = Encoding of auditory periodic stimuli; SLap = Sequence Learning with auditory periodic stimuli; Eaa = Encoding of auditory aperiodic stimuli; SLaa = Sequence learning with auditory aperiodic stimuli. ^a^ *r*. ^b^ Cohen’s *d*. * *p* < 0.05.

**Table 2:** Correlations between the sequencing tasks and time perception per language-based group and cross-group comparisons of the correlations (Fisher’s Z test). Values in bold indicate the main results, concerning both evidence in favor of the alternative (pro-TSF, beat, and aperiodic encoding) hypothesis and in favor of the null hypothesis (prediction-based, beat, and sequence learning plus duration and aperiodic encoding).

		Portuguese		Greek		Fisher’s Z (Z, *p*)
Domain	Sequencing Task	Beat	Duration	Beat	Duration	
Visual	Ev	0.228 ** 9.699	0.119 *0.530*	0.154 *0.557*	0.188 *0.825*	Z = −0.495, *p* = 0.31
	SLv	**−0.002** ***0.101***	−0.085 *0.053*	**0.084** ***0.288***	−0.116 *0.090*	---
Auditory periodic	Eap	−0.042 *0.072*	0.454 *** >100	0.168 *0.652*	0.275 * 2.976	Z = −1.335, *p* = 0.091
	Slap	0.218 ** 6.927	0.002 *0.105*	0.176 *0.713*	−0.075 *0.108*	Z = −0.281, *p* = 0.389
Auditory aperiodic	Eaa	0.450 *** ***>100***	**−0.069** ***0.059***	**0.165** ***0.629***	**−0.094** ***0.099***	Z = −2.048, *p* = 0.02 *
	SLaa	0.147 * 0.962	0.072 *0.240*	0.020 *0.181*	−0.066 *0.112*	Z = −0.824, *p* = 0.205

Note: BF10 values are indicated below correlations, in italic. Ev = Encoding visual stimuli; SLv = Sequence Learning with visual stimuli; Eap = Encoding of auditory periodic stimuli; SLap = Sequence Learning with auditory periodic stimuli; Eaa = Encoding of auditory aperiodic stimuli; SLaa = Sequence learning with auditory aperiodic stimuli. * *p* < 0.05, ** *p* < 0.01, and *** *p* < 0.001.

## Data Availability

The databases used in this study are available at the osf link: https://osf.io/gpe8h/?view_only=8ccc4903996c445fba0932dd493024cf (accessed on 10 June 2025).

## Supplementary Materials

The following supporting information can be downloaded at: https://www.mdpi.com/article/10.3390/brainsci15060642/s1.
