# Supplementary material for: Is It About Speech or About Prediction? Testing Between Two Accounts of the Rhythm–Reading Link
Source: Brain Sci. 2025 Jun 14;15(6):642. doi: 10.3390/brainsci15060642 (PMC12191367; doi:10.3390/brainsci15060642)
Supplement: Supplementary file 1 [file brainsci-15-00642-s001.zip › brainsci-3660690-supplementary.pdf]

**Supplementary Materials**

**Table S1** - Stimulus sequences for Encoding visual stimuli (Ev).

| | Sequence 1 | Sequence 2 | Answer | Length (number of letters) |
|---|---|---|---|---|
| 1 | MVRXVRXR | MVRXVRXV | Different | 8 |
| 2 | MVRXRRRM | MVRXRRRM | Same | 8 |
| 3 | MVRXSSSSV | MSVRXSSSV | Different | 9 |
| 4 | MSVRXSSSV | MVRXSSSSV | Different | 9 |
| 5 | MVRXRRRRR | MVRXRRRRR | Same | 9 |
| 6 | MSSSVRXRM | MSSSVRXRM | Same | 9 |
| 7 | VXSSVRXRRR | VXSVRXVRXM | Different | 10 |
| 8 | VXSVRXVRXM | VXSSVRXRRR | Different | 10 |
| 9 | VXVRXRRRRM | VXVRXRRRRM | Same | 10 |
| 10 | MSSVRXSSSV | MSSVRXSSSV | Same | 10 |
| 11 | MSVRXRRRRRM | MVRXSVRXRRM | Different | 11 |
| 12 | MSSSSSVRXVS | MSSSSSVRXRM | Different | 11 |
| 13 | VXSVRXVRXVS | MSVRXVRXSVS | Different | 11 |
| 14 | VXSVRXRRRRR | VXSVRXRRRRR | Same | 11 |
| 15 | VXVRXVRXRRR | VXVRXVRXRRR | Same | 11 |
| 16 | VXVRXSSVRXM | VXVRXSSVRXM | Same | 11 |
| 17 | VXVRXSSSSSVS | VXSSSSSVRXSV | Different | 12 |
| 18 | MVRXSSVRXRRR | MSSVRXVRXRRR | Different | 12 |
| 19 | MSSVRXVRXSVS | MSVRXSSVRXVS | Different | 12 |
| 20 | MVRXSVRXVRXR | VXVRXSVRXSSV | Different | 12 |
| 21 | MSVRXRRRRRRR | MSVRXRRRRRRR | Same | 12 |
| 22 | VXSSVRXVRXVS | VXSSVRXVRXVS | Same | 12 |
| 23 | VXSSVRXVRXRR | VXSSVRXVRXRR | Same | 12 |
| 24 | VXVRXVRXSSSV | VXVRXVRXSSSV | Same | 12 |

**Table S2** - Stimulus sequences for Sequence Learning with visual stimuli (SLv).

| | **Sequence 1** | **Answer** | **Length (number of letters)** |
|---|---|---|---|
| 1 | VXS**S** | Correct | 4 |
| 2 | VXR**X** | Incorrect | 4 |
| 3 | MSS**S** | Correct | 4 |
| 4 | MSS**V** | Incorrect | 4 |
| 5 | VXSS**S** | Correct | 5 |
| 6 | VXSV**S** | Incorrect | 5 |
| 7 | MSSS**V** | Correct | 5 |
| 8 | MSSV**X** | Incorrect | 5 |
| 9 | MVRXS**S** | Correct | 6 |
| 10 | MVRXR**X** | Incorrect | 6 |
| 11 | MSSSS**S** | Correct | 6 |
| 12 | MSSVR**S** | Incorrect | 6 |
| 13 | MSSVRXR**R** | Correct | 8 |
| 14 | MSSVRXV**X** | Incorrect | 8 |
| 15 | VXSVRXR**R** | Correct | 8 |
| 16 | VXSVRXS**X** | Incorrect | 8 |
| 17 | MVRXSSSS**S** | Correct | 9 |
| 18 | MVRXSSSV**X** | Incorrect | 9 |
| 19 | MSSSVRXR**R** | Correct | 9 |
| 20 | MSSSVRXS**X** | Incorrect | 9 |
| 21 | MSVRXVRXR**R** | Correct | 10 |
| 22 | VXVRXSSVR**S** | Incorrect | 10 |
| 23 | VXSSSVRXR**R** | Correct | 10 |
| 24 | VXSSSVRXS**X** | Incorrect | 10 |

The final letter is indicated in bold since it is the target letter.

**Table S3** - Stimulus sequences for Encoding of auditory periodic stimuli (Eap).

| | | Sequences | Answer | Length (number of syllables) |
|---|---|---|---|---|
| 1 | Sequence 1 | Ba-Gó-Mê-Mê-Mê-Ba-Pu-Gó | Different | 8 |
| | Sequence 2 | **Di-**Ba-Pu-Gó-Mê-Ba-Pu-Gó | | |
| 2 | Sequence 1 | Ba-Gó-Mê-Ba-Pu-Gó-Ba-Mê | Same | 8 |
| | Sequence 2 | **Ba-**Gó-Mê-Ba-Pu-Gó-Ba-Mê | | |
| 3 | Sequence 1 | Ba-Gó-Ba-Pu-Gó-Mê-Mê-Mê-Ba | Different | 9 |
| | Sequence 2 | Ba-Gó-**Mê-**Mê-Mê-Ba-Pu-Gó-Ba | | |
| 4 | Sequence 1 | Ba-Gó-Mê-Ba-Pu-Gó-Ba-Pu-Gó | Different | 9 |
| | Sequence 2 | Ba-Gó-**Ba-**Pu-Gó-Mê-Ba-Pu-Gó | | |
| 5 | Sequence 1 | Ba-Gó-Mê-Ba-Pu-Gó-Pu-Pu-Di | Same | 9 |
| | Sequence 2 | Ba-Gó-**Mê-**Ba-Pu-Gó-Pu-Pu-Di | | |
| 6 | Sequence 1 | Di-Mê-Mê-Mê-Ba-Pu-Gó-Ba-Mê | Same | 9 |
| | Sequence 2 | Di-Mê-**Mê-**Mê-Ba-Pu-Gó-Ba-Mê | | |
| 7 | Sequence 1 | Di-Ba-Pu-Gó-Ba-Pu-Gó-Pu-Pu-Di | Different | 10 |
| | Sequence 2 | Di-Ba-Pu-Gó-**Mê-**Ba-Pu-Gó-Pu-Di | | |
| 8 | Sequence 1 | Ba-Gó-Mê-Mê-Ba-Pu-Gó-Mê-Mê-Ba | Different | 10 |
| | Sequence 2 | Ba-Gó-**Ba-**Pu-Gó-Ba-Pu-Gó-Mê-Ba | | |
| 9 | Sequence 1 | Di-Mê-Mê-Ba-Pu-Gó-Pu-Pu-Pu-Di | Same | 10 |
| | Sequence 2 | Di-Mê-Mê-**Ba-**Pu-Gó-Pu-Pu-Pu-Di | | |
| 10 | Sequence 1 | Di-Ba-Pu-Gó-Ba-Pu-Gó-Mê-Mê-Ba | Same | 10 |
| | Sequence 2 | Di-Ba-**Pu-**Gó-Ba-Pu-Gó-Mê-Mê-Ba | | |
| 11 | Sequence 1 | Ba-Gó-Ba-Pu-Gó-Mê-Mê-Mê-Mê-Ba-Mê | Different | 11 |
| | Sequence 2 | **Di-**Mê-Ba-Pu-Gó-Mê-Mê-Mê-Mê-Ba-Mê | | |
| 12 | Sequence 1 | Di-Mê-Ba-Pu-Gó-Mê-Mê-Mê-Mê-Ba-Mê | Different | 11 |
| | Sequence 2 | **Ba-**Gó-Ba-Pu-Gó-Mê-Mê-Mê-Mê-Ba-Mê | | |
| 13 | Sequence 1 | Di-Mê-Ba-Pu-Gó-Ba-Pu-Gó-Mê-Ba-Mê | Different | 11 |
| | Sequence 2 | **Ba-**Gó-Mê-Ba-Pu-Gó-Ba-Pu-Gó-Ba-Mê | | |
| 14 | Sequence 1 | Ba-Gó-Mê-Mê-Ba-Pu-Gó-Pu-Pu-Pu-Pu | Same | 11 |
| | Sequence 2 | **Ba-**Gó-Mê-Mê-Ba-Pu-Gó-Pu-Pu-Pu-Pu | | |
| 15 | Sequence 1 | Di-Mê-Mê-Mê-Mê-Mê-Mê-Ba-Pu-Gó-Di | Same | 11 |
| | Sequence 2 | **Di-**Mê-Mê-Mê-Mê-Mê-Mê-Ba-Pu-Gó-Di | | |
| 16 | Sequence 1 | Ba-Gó-Mê-Ba-Pu-Gó-Mê-Ba-Pu-Gó-Di | Same | 11 |
| | Sequence 2 | **Ba-**Gó-Mê-Ba-Pu-Gó-Mê-Ba-Pu-Gó-Di | | |

| | | | | |
|---|---|---|---|---|
| 17 | Sequence 1 | Di-Ba-Pu-Gó-Mê-Ba-Pu-Gó-Pu-Pu-Pu-Pu | Differen t | 12 |
| | Sequence 2 | Di-Ba-Pu-Gó-Mê-**Mê-**Ba-Pu-Gó-Pu-Pu-Di | | |
| 18 | Sequence 1 | Di-Mê-Mê-Ba-Pu-Gó-Ba-Pu-Gó-Pu-Pu-Pu | Differen t | 12 |
| | Sequence 2 | Di-**Ba-**Pu-Gó-Mê-Mê-Ba-Pu-Gó-Pu-Pu-Pu | | |
| 19 | Sequence 1 | Di-Mê-Ba-Pu-Gó-Mê-Mê-Ba-Pu-Gó-Ba-Mê | Differen t | 12 |
| | Sequence 2 | Di-Mê-**Mê-**Ba-Pu-Gó-Ba-Pu-Gó-Mê-Ba-Mê | | |
| 20 | Sequence 1 | Ba-Gó-Mê-Mê-Ba-Pu-Gó-Mê-Ba-Pu-Gó-Ba | Differen t | 12 |
| | Sequence 2 | **Di-**Mê-Mê-Mê-Ba-Pu-Gó-Mê-Ba-Pu-Gó-Ba | | |
| 21 | Sequence 1 | Di-Mê-Mê-Mê-Mê-Ba-Pu-Gó-Pu-Pu-Pu-Di | Same | 12 |
| | Sequence 2 | Di-Mê-Mê-Mê-Mê-**Ba-**Pu-Gó-Pu-Pu-Pu-Di | | |
| 22 | Sequence 1 | Di-Mê-Mê-Mê-Ba-Pu-Gó-Ba-Pu-Gó-Ba-Mê | Same | 12 |
| | Sequence 2 | Di-**Mê-**Mê-Mê-Ba-Pu-Gó-Ba-Pu-Gó-Ba-Mê | | |
| 23 | Sequence 1 | Ba-Gó-Mê-Mê-Mê-Ba-Pu-Gó-Mê-Mê-Mê-Ba | Same | 12 |
| | Sequence 2 | Ba-Gó-**Mê-**Mê-Mê-Ba-Pu-Gó-Mê-Mê-Mê-Ba | | |
| 24 | Sequence 1 | Di-Mê-Mê-Ba-Pu-Gó-Mê-Ba-Pu-Gó-Pu-Di | Same | 12 |
| | Sequence 2 | **Di-**Mê-Mê-Ba-Pu-Gó-Mê-Ba-Pu-Gó-Pu-Di | | |

Syllables in bold indicate the ones that were changed in different versions.

**Table S4** - Stimulus sequences for Sequence Learning with auditory periodic stimuli (SLap).

| | **Sequence** | **Answer** | **Length (number of syllables)** |
|---|---|---|---|
| 1 | Di-Ba-Pu-**Gó** | Correct | 4 |
| 2 | Di-Ba-Pu-**Mê** | Incorrect | 4 |
| 3 | Ba-Gó-Pu-**Pu** | Correct | 4 |
| 4 | Ba-Gó-Mê-**Gó** | Incorrect | 4 |
| 5 | Ba-Gó-Mê-Mê-**Mê** | Correct | 5 |
| 6 | Ba-Gó-Mê-Ba-**Mê** | Incorrect | 5 |
| 7 | Di-Mê-Mê-Ba-**Mê** | Correct | 5 |
| 8 | Di-Ba-Pu-Gó-**Gó** | Incorrect | 5 |
| 9 | Di-Ba-Pu-Gó-Mê-**Mê** | Correct | 6 |
| 10 | Di-Ba-Pu-Gó-Pu-**Gó** | Incorrect | 6 |
| 11 | Di-Mê-Mê-Mê-Mê-**Mê** | Correct | 6 |
| 12 | Di-Mê-Mê-Ba-Pu-**Mê** | Incorrect | 6 |
| 13 | Di-Mê-Mê-Ba-Pu-Gó-Mê-**Ba** | Correct | 8 |
| 14 | Di-Mê-Mê-Ba-Pu-Gó-Pu-**Gó** | Incorrect | 8 |
| 15 | Ba-Gó-Mê-Mê-Mê-Mê-Mê-**Ba** | Correct | 8 |
| 16 | Ba-Gó-Mê-Mê-Mê-Mê-Ba-**Gó** | Incorrect | 8 |
| 17 | Di-Ba-Pu-Gó-Mê-Mê-Mê-Mê-**Ba** | Correct | 9 |
| 18 | Di-Ba-Pu-Gó-Mê-Mê-Ba-Pu-**Mê** | Incorrect | 9 |
| 19 | Di-Mê-Mê-Mê-Ba-Pu-Gó-Pu-**Pu** | Correct | 9 |
| 20 | Di-Mê-Mê-Mê-Ba-Pu-Gó-Ba-**Gó** | Incorrect | 9 |
| 21 | Ba-Gó-Ba-Pu-Gó-Mê-Mê-Mê-Mê-**Mê** | Correct | 10 |
| 22 | Ba-Gó-Mê-Ba-Pu-Gó-Mê-Ba-Pu-**Ba** | Incorrect | 10 |
| 23 | Ba-Gó-Ba-Pu-Gó-Ba-Pu-Gó-Pu-**Pu** | Correct | 10 |
| 24 | Ba-Gó-Ba-Pu-Gó-Ba-Pu-Gó-Ba-**Gó** | Incorrect | 10 |

The final syllable is indicated in bold since it is the target syllable.

**Table S5** - Stimulus sequences for Encoding of auditory aperiodic stimuli (Eaa).

| | | **Sequences** | **Inter-onset-intervals across syllables** | **Answer** | **Length (number of syllables)** |
|---|---|---|---|---|---|
| 1 | Sequence 1 | Ba-Gó-Mê-Mê-Mê-Ba-Pu-Gó | 350-*550*-350-350-350-*550*-350-350 | Different | 8 |
| | Sequence 2 | **Di-**Ba-Pu-Gó-Mê-Ba-Pu-Gó | | | |
| 2 | Sequence 1 | Ba-Gó-Mê-Ba-Pu-Gó-Ba-Mê | 350-*550*-350-350-350-*550*-350-350 | Same | 8 |
| | Sequence 2 | **Ba-**Gó-Mê-Ba-Pu-Gó-Ba-Mê | | | |
| 3 | Sequence 1 | Ba-Gó-Ba-Pu-Gó-Mê-Mê-Mê-Ba | *550*-350-350-350-*600*-350-350-350-350 | Different | 9 |
| | Sequence 2 | Ba-Gó-**Mê-**Mê-Mê-Ba-Pu-Gó-Ba | | | |
| 4 | Sequence 1 | Ba-Gó-Mê-Ba-Pu-Gó-Ba-Pu-Gó | *550*-350-350-350-*600*-350-350-350-350 | Different | 9 |
| | Sequence 2 | Ba-Gó-**Ba-**Pu-Gó-Mê-Ba-Pu-Gó | | | |
| 5 | Sequence 1 | Ba-Gó-Mê-Ba-Pu-Gó-Pu-Pu-Di | *550*-350-350-350-*600*-350-350-350-350 | Same | 9 |
| | Sequence 2 | Ba-Gó-**Mê-**Ba-Pu-Gó-Pu-Pu-Di | | | |
| 6 | Sequence 1 | Di-Mê-Mê-Mê-Ba-Pu-Gó-Ba-Mê | *550*-350-350-350-*600*-350-350-350-350 | Same | 9 |
| | Sequence 2 | Di-Mê-**Mê-**Mê-Ba-Pu-Gó-Ba-Mê | | | |
| 7 | Sequence 1 | Di-Ba-Pu-Gó-Ba-Pu-Gó-Pu-Pu-Di | 350-350-*550*-350-350-*650*-350-350-350-350 | Different | 10 |
| | Sequence 2 | Di-Ba-Pu-Gó-**Mê-**Ba-Pu-Gó-Pu-Di | | | |
| 8 | Sequence 1 | Ba-Gó-Mê-Mê-Ba-Pu-Gó-Mê-Mê-Ba | 350-350-*550*-350-350-*650*-350-350-350-350 | Different | 10 |
| | Sequence 2 | Ba-Gó-**Ba-**Pu-Gó-Ba-Pu-Gó-Mê-Ba | | | |
| 9 | Sequence 1 | Di-Mê-Mê-Ba-Pu-Gó-Pu-Pu-Pu-Di | 350-350-*550*-350-350-*650*-350-350-350-350 | Same | 10 |
| | Sequence 2 | Di-Mê-Mê-**Ba-**Pu-Gó-Pu-Pu-Pu-Di | | | |
| 10 | Sequence 1 | Di-Ba-Pu-Gó-Ba-Pu-Gó-Mê-Mê-Ba | 350-350-*550*-350-350-*650*-350-350-350-350 | Same | 10 |
| | Sequence 2 | Di-Ba-**Pu-**Gó-Ba-Pu-Gó-Mê-Mê-Ba | | | |
| 11 | Sequence 1 | Ba-Gó-Ba-Pu-Gó-Mê-Mê-Mê-Mê-Ba-Mê | 350-350-350-*600*-350-350-*550*-350-350-*450*-350 | Different | 11 |
| | Sequence 2 | Mê-Mê-Mê-Mê-Ba-Mê | | | |
| 12 | Sequence 1 | Di-Mê-Ba-Pu-Gó-Mê-Mê-Mê-Mê-Ba-Mê | 350-350-350-*600*-350-350-*550*-350-350-*450*-350 | Different | 11 |
| | Sequence 2 | **Ba-**Gó-Ba-Pu-Gó-Mê-Mê-Mê-Mê-Ba-Mê | | | |

| | | | | | |
|---|---|---|---|---|---|
| 13 | Sequence 1 | Di-Mê-Ba-Pu-Gó-Ba-Pu-Gó-Mê-Ba-Mê | 350-350-350-*600*-350-350-*550*-350-350-*450*-350 | Different | 11 |
| | Sequence 2 | **Ba-**Gó-Mê-Ba-Pu-Gó-Ba-Pu-Gó-Ba-Mê | | | |
| 14 | Sequence 1 | Ba-Gó-Mê-Mê-Ba-Pu-Gó-Pu-Pu-Pu-Pu | 350-350-350-*600*-350-350-*550*-350-350-*450*-350 | Same | 11 |
| | Sequence 2 | **Ba-**Gó-Mê-Mê-Ba-Pu-Gó-Pu-Pu-Pu-Pu | | | |
| 15 | Sequence 1 | Di-Mê-Mê-Mê-Mê-Mê-Mê-Ba-Pu-Gó-Di | 350-350-350-*600*-350-350-*550*-350-350-*450*-350 | Same | 11 |
| | Sequence 2 | **Di-**Mê-Mê-Mê-Mê-Mê-Mê-Ba-Pu-Gó-Di | | | |
| 16 | Sequence 1 | Ba-Gó-Mê-Ba-Pu-Gó-Mê-Ba-Pu-Gó-Di | 350-350-350-*600*-350-350-*550*-350-350-*450*-350 | Same | 11 |
| | Sequence 2 | **Ba-**Gó-Mê-Ba-Pu-Gó-Mê-Ba-Pu-Gó-Di | | | |
| 17 | Sequence 1 | Di-Ba-Pu-Gó-Mê-Ba-Pu-Gó-Pu-Pu-Pu-Pu | *550*-350-*450*-350-350-350-350-*550*-*450*-350-350-350 | Different | 12 |
| | Sequence 2 | Di-Ba-Pu-Gó-Mê-**Mê-**Ba-Pu-Gó-Pu-Pu-Di | | | |
| 18 | Sequence 1 | Di-Mê-Mê-Ba-Pu-Gó-Ba-Pu-Gó-Pu-Pu-Pu | *550*-350-*450*-350-350-350-350-*550*-*450*-350-350-350 | Different | 12 |
| | Sequence 2 | Di-**Ba-**Pu-Gó-Mê-Mê-Ba-Pu-Gó-Pu-Pu-Pu | | | |
| 19 | Sequence 1 | Di-Mê-Ba-Pu-Gó-Mê-Mê-Ba-Pu-Gó-Ba-Mê | *550*-350-*450*-350-350-350-350-*550*-*450*-350-350-350 | Different | 12 |
| | Sequence 2 | Di-Mê-**Mê-**Ba-Pu-Gó-Ba-Pu-Gó-Mê-Ba-Mê | | | |
| 20 | Sequence 1 | Ba-Gó-Mê-Mê-Ba-Pu-Gó-Mê-Ba-Pu-Gó-Ba | *550*-350-*450*-350-350-350-350-*550*-*450*-350-350-350 | Different | 12 |
| | Sequence 2 | **Di-**Mê-Mê-Mê-Ba-Pu-Gó-Mê-Ba-Pu-Gó-Ba | | | |
| 21 | Sequence 1 | Di-Mê-Mê-Mê-Mê-Ba-Pu-Gó-Pu-Pu-Pu-Di | *550*-350-*450*-350-350-350-350-*550*-*450*-350-350-350 | Same | 12 |
| | Sequence 2 | Di-Mê-Mê-Mê-Mê-**Ba-**Pu-Gó-Pu-Pu-Pu-Di | | | |
| 22 | Sequence 1 | Di-Mê-Mê-Mê-Ba-Pu-Gó-Ba-Pu-Gó-Ba-Mê | *550*-350-*450*-350-350-350-350-*550*-*450*-350-350-350 | Same | 12 |
| | Sequence 2 | Di-**Mê-**Mê-Mê-Ba-Pu-Gó-Ba-Pu-Gó-Ba-Mê | | | |
| 23 | Sequence 1 | Ba-Gó-Mê-Mê-Mê-Ba-Pu-Gó-Mê-Mê-Mê-Ba | *550*-350-*450*-350-350-350-350-*550*-*450*-350-350-350 | Same | 12 |
| | Sequence 2 | Ba-Gó-**Mê-**Mê-Mê-Ba-Pu-Gó-Mê-Mê-Mê-Ba | | | |
| 24 | Sequence 1 | Di-Mê-Mê-Ba-Pu-Gó-Mê-Ba-Pu-Gó-Pu-Di | *550*-350-*450*-350-350-350-350-*550*-*450*-350-350-350 | Same | 12 |
| | Sequence 2 | **Di-**Mê-Mê-Ba-Pu-Gó-Mê-Ba-Pu-Gó-Pu-Di | | | |

Syllables in bold indicate the ones that were changed in different versions and values in italics represent the non-isochronous onsets.

**Table S6** - Stimulus sequences for Sequence Learning with auditory aperiodic stimuli (SLaa).

| | **Sequence** | **Inter-onset-intervals across syllables** | **Answer** | **Length (number of syllables)** |
|---|---|---|---|---|
| 1 | Di-Mê-Mê-**Mê** | 350-*550*-350-350 | Correct | 4 |
| 2 | Di-Ba-Pu-**Mê** | 350-*550*-350-350 | Incorrect | 4 |
| 3 | Di-Mê-Mê-**Mê** | 350-*550*-350-350 | Correct | 4 |
| 4 | Di-Mê-Ba-**Mê** | 350-*550*-350-350 | Incorrect | 4 |
| 5 | Ba-Gó-Mê-Mê-**Mê** | *500*-350-*450*-350-350 | Correct | 5 |
| 6 | Ba-Gó-Mê-Ba-**Mê** | *500*-350-*450*-350-350 | Incorrect | 5 |
| 7 | Ba-Gó-Mê-Mê-**Ba** | *500*-350-*450*-350-350 | Correct | 5 |
| 8 | Di-Mê-Ba-Pu-**Mê** | *500*-350-*450*-350-350 | Incorrect | 5 |
| 9 | Di-Ba-Pu-Gó-Mê-**Ba** | 350-350-*550*-*450*-350-350 | Correct | 6 |
| 10 | Di-Ba-Pu-Gó-Ba-**Mê** | 350-350-*550*-*450*-350-350 | Incorrect | 6 |
| 11 | Ba-Gó-Mê-Mê-Mê-**Ba** | 350-350-*550*-*450*-350-350 | Correct | 6 |
| 12 | Ba-Gó-Mê-Mê-Ba-**Gó** | 350-350-*550*-*450*-350-350 | Incorrect | 6 |
| 13 | Di-Mê-Ba-Pu-Gó-Mê-Mê-**Ba** | 350-*500*-*500*-350-*450*-350-350-350 | Correct | 8 |
| 14 | Di-Mê-Mê-Ba-Pu-Gó-Pu-**Gó** | 350-*500*-*500*-350-*450*-350-350-350 | Incorrect | 8 |
| 15 | Ba-Gó-Mê-Ba-Pu-Gó-Pu-**Pu** | 350-*500*-*500*-350-*450*-350-350-350 | Correct | 8 |
| 16 | Ba-Gó-Mê-Ba-Pu-Gó-Ba-**Gó** | 350-*500*-*500*-350-*450*-350-350-350 | Incorrect | 8 |
| 17 | Di-Ba-Pu-Gó-Ba-Pu-Gó-Mê-**Ba** | 350-*550*-350-*450*-*450*-350-*400*-350-350 | Correct | 9 |
| 18 | Di-Ba-Pu-Gó-Ba-Pu-Gó-Pu-**Gó** | 350-*550*-350-*450*-*450*-350-*400*-350-350 | Incorrect | 9 |
| 19 | Di-Ba-Pu-Gó-Ba-Pu-Gó-Pu-**Pu** | 350-*550*-350-*450*-*450*-350-*400*-350-350 | Correct | 9 |
| 20 | Di-Ba-Pu-Gó-Ba-Pu-Gó-Mê-**Gó** | 350-*550*-350-*450*-*450*-350-*400*-350-350 | Incorrect | 9 |
| 21 | Di-Mê-Ba-Pu-Go-Mê-Mê-Mê-Mê-**Mê** | *400*-*550*-350-350-*450*-350-350-350-*500*-350 | Correct | 10 |
| 22 | Di-Mê-Ba-Pu-Gó-Mê-Mê-Ba-Pu-**Mê** | *400*-*550*-350-350-*450*-350-350-350-*500*-350 | Incorrect | 10 |
| 23 | Ba-Gó-Mê-Mê-Mê-Ba-Pu-Gó-Pu-**Pu** | *400*-*550*-350-350-*450*-350-350-350-*500*-350 | Correct | 10 |
| 24 | Ba-Gó-Mê-Mê-Mê-Ba-Pu-Gó-Ba-**Gó** | *400*-*550*-350-350-*450*-350-350-350-*500*-350 | Incorrect | 10 |

The final syllable is indicated in bold since it is the target syllable and values in italics represent the non-isochronous onsets.

**Table S7** - Time structure of beep sequences used in the duration perception task (ms)

| Type | Interval 1 | Interval 2 | Difference | Type | Interval 1 | Interval 2 | Difference |
|---|---|---|---|---|---|---|---|
| Slow down | 300 | 433 | -133 | Speed up | 433 | 300 | 133 |
| Slow down | 167 | 300 | -133 | Speed up | 300 | 167 | 133 |
| Slow down | 433 | 467 | -34 | Speed up | 467 | 433 | 34 |
| Slow down | 167 | 733 | -566 | Speed up | 733 | 167 | 566 |
| Slow down | 300 | 467 | -167 | Speed up | 467 | 300 | 167 |
| Slow down | 134 | 434 | -301 | Speed up | 433 | 134 | 299 |
| Slow down | 233 | 534 | -301 | Speed up | 534 | 233 | 301 |
| Slow down | 433 | 500 | -67 | Speed up | 500 | 433 | 67 |
